# The Popular Science Monthly

**Published:** 1877-09

**Authors:** 


The Popular Science Monthly for September opens with another able original paper, by Herbert Spencer, on the development of the domestic relations, in which he indicates some of the most important lines of future social progress. The next article, "Odd Forms among Fishes," is by the late Prof. Sanborn Tenney, who, with the aid of numerous illustrations, gives a very entertaining account of sundry curious divergencies from the typical pattern in this division of animal life. The Observatories of Italy, of which there are no less than ten, under the patronage of the Government, are briefly described in the third article, with the work that each is doing. "On Drops," is a short but fully illustrated account of some remarkable experiments, showing, by the aid of electric illumination, the curious shapes which drops of fluid take on striking a hard surface. "Civilization and Morals," by our fellow citizen, Mr. J. N. Larned, is an instructive discussion of man's various relationships, and how out of these has grown up his present system of morals. The eighth article, "Instinct and Intelligence," by W. K. Brooks, of Johns Hopkins University, is of great interest, as tending to show that the distinction hitherto erected between men and animals in this regard has no actual existence in Nature. Among the five other papers that go to make up the body of the Magazine, all of which will fully repay the reader, there is a short but incisive article on "The Labor-Question," written before, but bearing directly on the recent strikes; and a sketch, with portrait, of Prof. Simon Newcomb, the distinguished Director of the U. S. Naval Observatory at Washington, and the present President of the American Association for the advancement of Science. The departments including Correspondence and Editor's Table are, as usual, full of interest and instruction. They contain pointed discussions of current scientific questions, notices of the latest scientific books, and, in the Popular Miscellany, brief but clearly-written abstracts of recent papers, and descriptions of new discoveries from the principal centres of scientific activity both at home and abroad.

